# Barriers to adherence to endotracheal tube suctioning guidelines among intensive care nurses at a Tanzanian national hospital: A qualitative study

**DOI:** 10.1371/journal.pone.0347186

**Published:** 2026-06-16

**Authors:** Loema Moshi Buyi, Rashid Heri, Menti Lastone Ndile

**Affiliations:** 1 Dodoma Regional Referral Hospital, Dodoma, Tanzania; 2 Department of Nursing Education and Management, School of Nursing, Muhimbili University of Health and Allied Sciences, Dar es Salaam, Tanzania; 3 Department of Clinical Nursing, School of Nursing, Muhimbili University of Health and Allied Sciences, Dar es Salaam, Tanzania; Public Library of Science, UNITED STATES OF AMERICA

## Abstract

**Background:**

Endotracheal tube suctioning (ETS) is a critical procedure for mechanically ventilated patients. Evidence-based guidelines exist to ensure its safe performance; however, adherence remains suboptimal, especially in resource-limited settings. This study aimed to explore the barriers to ETS guideline adherence among ICU nurses at Muhimbili National Hospital (MNH), Tanzania.

**Methods:**

An exploratory qualitative study was conducted in the medical and surgical ICUs of MNH. Seventeen ICU nurses with ≥1 year of experience were purposively sampled. Semi-structured interviews were performed, audio-recorded, transcribed verbatim, and analyzed using inductive thematic analysis following Braun and Clarke’s framework.

**Results:**

Three key barriers emerged: (1) Resource scarcity, including critical shortages of staff, equipment, and supplies; (2) Human and behavioral challenges, such as knowledge deficits and resistance to change away from routine practice; and (3) Guideline accessibility and usability, concerning poor accessibility and a perception that guidelines were not tailored to the local context.

**Conclusion:**

Non-adherence to ETS guidelines primarily reflects systemic constraints rather than individual neglect. Strengthening adherence requires multi-level interventions: improving staffing and resources, providing continuous training, and adapting international guidelines to the local context.

## Background

Endotracheal tube suctioning (ETS) is a frequently performed invasive nursing procedure in intensive care units (ICUs) and plays a critical role in maintaining airway patency, optimizing gas exchange, and preventing complications such as atelectasis and ventilator-associated pneumonia (VAP) [1]. As a core ICU nursing competency, ETS requires consistent adherence to evidence-based practices supported by ongoing training and access to clinical guidelines.

When ETS is performed incorrectly, it can result in significant adverse outcomes, including hypoxemia, cardiovascular instability, airway trauma, mucosal bleeding, and an increased risk of nosocomial infections [2]. To mitigate these risks, evidence-based ETS guidelines have been developed, emphasizing appropriate patient assessment, correct catheter selection, strict aseptic technique, and continuous patient monitoring throughout the procedure [3–5].

Despite the availability of these guidelines, adherence to recommended ETS practices remains suboptimal, particularly in low-resource settings. Multiple interacting barriers, including systemic constraints such as high nurse-to-patient ratios, shortages of equipment and consumables, and irregular access to clinical guidelines and in-service training influence nurses’ adherence. Behavioural factors, including reliance on habitual practices and risk perception, as well as contextual influences such as unit culture and limited supervisory support, further contribute to suboptimal adherence to ETS recommendations [6–12]. In contrast, non-adherence in well-resourced settings is more commonly attributed to time pressure or individual clinical judgment rather than resource limitations [10,13].

Evidence from Tanzania highlights these systemic challenges. A survey mapping emergency and critical care services conducted in ten districts and tertiary hospitals reported that only 20% of surveyed hospitals had staff trained in critical care, 40% had mechanical ventilators, and 44% had access to critical care practice guidelines [14]. These findings underscore significant gaps in both resources and training. Similarly, a study conducted in a tertiary hospital in Tanzania found that approximately 103 (80%) nurses had inadequate knowledge of evidence-based ETS practices, resulting in frequent deviations from standard care [15].

Poor adherence to ETS guidelines is associated with increased rates of VAP, prolonged ICU length of stay, and higher ICU mortality [3,16,17]. In resource-constrained ICUs, these largely preventable complications impose substantial clinical and economic burdens. Enhancing adherence to ETS guidelines, therefore, represents a feasible, low-cost opportunity to improve patient safety and clinical outcomes.

Although ETS non-adherence has been widely documented, the context-specific barriers influencing ICU nurses’ adherence in Tanzania remain insufficiently understood. A qualitative exploratory approach is therefore appropriate to capture nurses’ experiences and perceptions and to elucidate how systemic and contextual factors shape ETS practice. This study aims to explore the barriers to ICU nurses’ adherence to ETS guidelines at Muhimbili National Hospital (MNH) to inform contextually appropriate interventions and support safer ICU care.

## Methods and materials

### Study design

This exploratory qualitative study sought to gain in-depth insights into the barriers ICU nurses face in implementing ETS guidelines for mechanically ventilated patients. The approach was deemed appropriate as it allowed exploration of complex, context-specific phenomena through participants’ own experiences [18]. To ensure methodological rigour and transparent reporting, this study was conducted in accordance with the Standards for Reporting Qualitative Research (SRQR) checklist. The completed SRQR checklist is available as Supporting Information (S1 Checklist).

### Study setting

The study was conducted in the medical and surgical intensive care units of MNH. This national referral hospital serves a diverse patient population and is staffed by a multidisciplinary team of healthcare providers. The medical and surgical units collectively accommodate 34 beds and are supported by approximately 60 nurses, with educational qualifications ranging from diplomas to master’s levels.

### Participant recruitment and sample size

Nurses were purposively sampled [19] from the medical and surgical ICUs, with a minimum of one year of clinical experience as an inclusion criterion. The final sample size was guided by the principle of data saturation [20]. While the initial study design planned for ten interviews, a total of seventeen were conducted. Saturation was declared when additional interviews provided no new insights or themes pertinent to the study’s aims. All nurses who were purposively sampled agreed to participate and completed the interviews; there were no refusals or dropouts.

### Data collection tools and procedures

This study was conducted from May 15 to June 16, 2023. Data were collected using a semi-structured interview guide developed by the researchers after a comprehensive literature review on the topic. The final guide included three core open-ended questions, supplemented by probes to encourage deeper reflection (Supporting Information, S2 Text). Key questions included: *“Can you describe your typical experience when performing endotracheal suctioning on a patient in the ICU?”* and *“What factors or situations have made it difficult for you to apply endotracheal tube suctioning guidelines in your practice?”*

Eligible participants were fully informed of the study’s purpose and confidentiality measures before being invited to participate. After providing verbal consent, participants gave written consent on the day of their interview.

Interviews were conducted in Kiswahili, Tanzania’s national language, by a researcher fluent in both Kiswahili and English. They were held in a designated hospital room at a time convenient for the participant and lasted 40–60 minutes. All interviews were audio-recorded with the participant’s consent. Additionally, field notes were taken during each interview and served to contextualize the audio-recorded verbal responses. They primarily captured nonverbal cues (e.g., pauses, tone of voice, body language) and the interviewer’s immediate observations about the interview context. These notes were integrated with the verbatim transcripts during the analysis phase to provide a richer, more nuanced understanding of the participants’ responses and the overall meaning of the data.

### Data Analysis

Data were analyzed using an inductive thematic approach, guided by Braun and Clarke’s six-step framework [21]. The analysis began with the authors repeatedly reading the transcripts to familiarize themselves with the data. Meaningful text segments were then identified and assigned descriptive codes in Kiswahili to preserve their original meaning. To facilitate collaborative analysis, these codes were translated into English, with translation accuracy verified by bilingual team members to mitigate misinterpretation.

Through an iterative process, codes with similar patterns were grouped to generate initial themes and subthemes. This phase involved rigorous discussion among three co-authors (LB, MN, RH) and an external qualitative expert to ensure analytical consistency and reduce potential bias. The tentative thematic framework was then critically re-examined against the full dataset and refined through constant comparison to ensure it coherently and authentically represented the data. Any disagreements arising during coding or theme development were resolved through discussion until a shared consensus was reached among the research team. The final analysis yielded three overarching themes and six subthemes, each supported by representative quotes from participants. The themes and subthemes were selected because they clearly captured the most consistent and meaningful patterns across the data. They were coherent, distinct, and effectively reflected issues described by participants.

### Trustworthiness of the Study

To ensure the trustworthiness of the qualitative data, this study was evaluated against the four criteria proposed by Lincoln and Guba [22]: credibility, dependability, confirmability, and transferability.

Credibility was enhanced through multiple strategies. The interview guide was carefully designed and tailored to participants’ experiences. Member checking was conducted by sharing interview summaries with some participants to confirm the accuracy of the data and the researcher’s interpretive claims. Prolonged engagement in the research setting (before, during and after interviews) allowed the researcher to build rapport and gain a deeper understanding of contextual nuances. Peer debriefing with an external qualitative expert further challenged assumptions and strengthened the validity of findings.

Dependability and Confirmability were supported by employing a standardized, rigorously developed interview guide across all participants, ensuring consistency in data collection. A comprehensive audit trail was maintained, documenting data collection steps and analytical procedures. This transparency enables replication and demonstrates that findings are firmly grounded in participants’ narratives rather than researcher bias. Reflexive journaling was also used to bracket personal assumptions and mitigate potential researcher bias

Transferability was facilitated by providing thick, contextual descriptions of the study setting, detailed demographic profiles of participants and a transparent account of the analytic process. This enables readers to assess the applicability and relevance of the findings to similar clinical and cultural contexts.

### Ethical considerations

Ethical approval for this study was granted by the Institutional Review Board of Muhimbili University of Health and Allied Sciences (MUHAS; Ref. No: DA.282/298/01.C/1645) and the management of Muhimbili National Hospital (Ref. No: MNH/CRTCU/Perm/2023/315). Prior to each interview, the principal researcher obtained written informed consent. The study’s purpose and confidentiality measures were thoroughly explained, and participants were assured of their right to withdraw or decline to answer any question at any time. This process ensured that all participation was voluntary and transparent.

## Results

### Background characteristics of the participants

The study included 17 ICU nursing professionals with diverse backgrounds. Participants ranged in age from 26 to 47 years, with a mean age of approximately 34 years. The group was almost evenly split by sex, comprising 9 females and 8 males. Professionally, 9 were Nursing Officers (NOs), holding bachelor’s degrees and above, and 8 were Assistant Nursing Officers (ANOs), holding diplomas. Their ICU experience ranged from 1 to 9 years, with an average of about 4 years (Table 1).

**Table 1:** Demographic characteristics of participants (N = 17).

Characteristic	Details	n (%)
**Age (years)**	Range	26–47
	Mean (Average)	33.9
**Sex**	Female	9 (52.9%)
	Male	8 (47.1%)
**Educational level**	Diploma	8 (47.1%)
	Bachelor’s degree and above	9 (52.9%)
**Designation**	Nursing Officer (NO)	9 (52.9%)
	Assistant Nursing Officer (ANO)	8 (47.1%)
**ICU experience (years)**	Range	1–9
	Mean (Average)	4.0

### Themes, sub-themes, and codes

Three major themes emerged from the data analysis. Theme 1: Resource scarcity encompassed two subthemes: inadequate staffing and shortage of essential equipment and supplies. Theme 2: Human and behavioural challenges included two subthemes, namely knowledge deficit and resistance to change. Theme 3: Guideline accessibility and usability comprised two subthemes: accessibility issues and usability challenges. The themes, subthemes and corresponding codes are presented in Table 2. Representative quotations are provided in the Results section, and additional de-identified quotations supporting themes are available in S3 Table.

**Table 2:** Themes, sub-themes, and codes.

Themes	Subthemes	Codes
Resource scarcity	Inadequate staffing	Inadequate nurse numbers Excessive workload limits guideline adherence
	Shortage of essential equipment and supplies	Limited suction machines Limited suction catheters Insufficient oxygen cylinders
Human & behavioural challenges	Knowledge deficit	Insufficient training opportunities Lack of refresher courses
	Resistance to change	Reliance on routine practice Dependence on past experience over updated evidence Low motivation to adopt guidelines
Guideline accessibility & usability	Accessibility issues	Guidelines not posted in wards Not distributed to staff Difficult to locate
	Usability challenges	Implementation constrained by staff shortages Implementation requires uninterrupted supply of equipment/consumables

### Theme 1: Resource scarcity

Resource scarcity emerged as the most critical and overarching barrier to providing safe, guideline-adherent care during ETS. This theme is illustrated through two subthemes: 1) Inadequate staffing and 2) shortage of essential equipment and supplies.

#### Inadequate staffing.

A shortage of staff emerged as a critical barrier that undermines adherence to ETS guidelines. Nurses often find themselves responsible for multiple patients simultaneously, making it impractical to follow protocols as recommended. As one participant explained:

*"The guide says one, two, three… but when I’m alone with four patients, how will I implement this guide?"* (P6).

Similarly, another participant highlighted the mismatch between staffing ratios and guideline requirements:

*"With 18 beds and 6 staff, while guidelines require two providers per patient… this is usually impossible to follow."* (P3)

The shortage of staff was also linked to adverse patient outcomes, as noted by one participant:

*"If staffing is adequate, issues like ventilator-associated pneumonia wouldn’t be prevalent."* (P13)

*Shortage of essential equipment and supplies*The lack of basic supplies further hampers safe practice, compelling nurses to resort to substandard alternatives that may increase the risk of infection. For instance, one participant stated:

*"We usually don’t have enough supplies… so we use the suction catheters on our patients for an extended time."* (P9)

Similarly, the limited availability of suction machines often forces multiple patients to share equipment:

*"We have one machine shared among three patients. The only thing we can change is the suction tube and suction catheter."* (P13)

The situation becomes particularly critical when caring for infectious patients:

*"Sometimes we have only two suction machines and three infectious patients."* (P2)

In addition, faulty infrastructure and poorly maintained equipment further complicate care delivery. As two participants described:

*"Portable suction machines lack wheels; wall sockets fail. We waste time finding cables instead of focusing on patients."* (P3).

*“Sometimes there are only four oxygen cylinders for this entire building... this is a big issue.”* (P5)

### Theme 2: Human & behavioural challenges

Beyond resource constraints, the most significant barriers to implementing the ETS guideline are human and behavioral: namely, knowledge deficits and resistance to change.

#### Knowledge deficit.

Participants expressed limited confidence in their ability to correctly follow all the items in the ETS guideline during suctioning. A recurring concern was the lack of structured training, either formal orientation or on-the-job programs, leaving many reliant on informal learning from colleagues. As one participant explained:

*"I do not know what is in the guidelines as I have not been trained to use it, only through experience from my colleagues."* (P11)

Another participant highlighted the variability in practice resulting from the lack of standardized training, stating:

*"Someone instructed by one person and someone by another will perform suction differently."* (P 5)

Participants strongly underscored the need for structured and continuous education to strengthen competence and standardize practice:

*"Additional training is essential to enhance adherence to the guideline and deliver the best possible care to our patients."* (P17)

#### Resistance to change.

Resistance to the ETS guideline often stems from valuing long-standing routines over updated evidence, which fosters a culture that discourages adherence to evidence-based protocols. As participants reflected:

*“We do things out of routine because we used to do it that way....”* (P17)

*“I’ve suctioned for 10 years…what can you teach me?”* (P9)

Some nurses acknowledged that even when resources and guidelines are available, deeply ingrained habits and the realities of practice make strict adherence difficult. As one participant noted:

*"…strictly following guidelines is very difficult because it increases tasks more than when you do it the way we are used to"* (P8)

### Theme 3: Guideline Accessibility & Usability

The effective integration of clinical guidelines into daily practice is hindered by several barriers that extend far beyond simple awareness. The challenges are related to issues of accessibility and practical usability.

#### Accessibility issues.

When guidelines are physically difficult to locate or obtain, this prevents staff from referencing them when needed. This is a common concern. As one participant argued:

*"They need to be prominently displayed at the nursing station, not just posted on a random notice board where they are easily missed.”* (P6)

This lack of strategic access forces staff to rely on inefficient methods, making compliance a persistent challenge. One participant expressed:

"*Relying on individual initiative to look up guidelines on a personal phone or consult a busy nurse manager is an obstacle to ensuring adherence to protocols."* (P12)

#### Usability challenges.

A significant barrier to implementation is the failure to adapt guidelines to the specific context of care. When guidelines are not tailored to local staffing ratios, resource availability, and environmental constraints, they are perceived as theoretical rather than practical tools. This sentiment was captured by a participant who noted,

*"Our situation isn’t ideal for full implementation ETS guideline. For example, requiring two nurses per patient or an unlimited supply of sterile kits isn’t always possible here."* (P10)

## Discussion

This study identified three primary thematic barriers to the consistent implementation of ETS guidelines: (1) resource scarcity, (2) human and behavioural challenges, and (3) issues of guideline accessibility and usability. While these barriers have been reported in previous studies, the present findings illustrate how their strong interdependence and consequences are uniquely amplified within the Tanzanian ICU context, where systemic constraints shape everyday clinical decision-making. Importantly, non-adherence to the implementation of ETS guidelines in this setting is not simply an issue of neglect but rather a response to structural limitations.

Resource scarcity emerged as the most dominant barrier. While global literature cites staffing shortages as a barrier to guideline implementation [23,24], studies from well-resourced settings often describe understaffing as an episodic issue. This stands in stark contrast to the persistent, structural deficits described in the present study, where nurse-to-patient ratios routinely fall far below the staffing levels required by ETS recommendations. This divergence is critical, as guidelines that presume the availability of multiple nurses per procedure become fundamentally incompatible with local practice realities. Similar associations between workload and non-adherence have been reported elsewhere [25,26]. yet in the Tanzanian context, high workload is currently a fixed constraint, thereby rendering some guideline elements functionally unattainable rather than selectively ignored.

Furthermore, while studies demonstrate that adequate staffing in high-income settings reduces ventilator-associated pneumonia and other hospital-acquired infections [27–29], The present findings suggest a more troubling implication in low-resource contexts where the absence of staffing itself becomes a direct driver of preventable harm [30].

Equipment and supply shortages further highlighted important contextual differences. Participants in this study described the routine unavailability of essential equipment and consumables, often necessitating reuse or sharing of items between patients. Such practices are rarely reported in studies from higher-resource settings [31,32] which characterizes this practice as an exception or deviation from standard rather than a survival strategy adopted in response to resource constraints. While the WHO Global Patient Safety Report emphasizes that essential equipment is a prerequisite for safe care [33], this study reveals how that prerequisite remains unmet in practice.

Human and behavioural barriers in this study were also significant. Knowledge deficits and limited evidence-based practice (EBP) training are frequently attributed to individual shortcomings or a lack of motivation [24,34]. However, in the Tanzanian context, these deficits were clearly linked to the inadequate institutionalized training mechanisms, forcing nurses to rely on informal, peer-led learning. This may contrast with settings where formal continuing professional development exists but may be underutilized. The expressed eagerness for training among participants challenges assumptions that resistance to guidelines reflects attitudinal barriers and instead points to missed institutional opportunities for capacity building.

Similarly, resistance to change, which is often characterized as a behavioural or cultural obstacle, emerged here as a contextually rational response. Long-standing practices were not maintained out of disregard for evidence but because they aligned more closely with available resources and staffing patterns. When guidelines are seen as adding workload without providing additional support, they risk being dismissed as unrealistic. The findings suggest that, when feasibility constraints and contextual realities are not adequately addressed, efforts to promote EBP risk reinforcing disengagement rather than improving adherence [35,36].

Finally, issues of guideline accessibility and usability further illustrate the limitations of applying globally developed recommendations without contextual adaptation. While previous studies advocate for better dissemination of guidelines at the point of care [35]. this study demonstrates that visibility alone is insufficient when guideline assumptions conflict with staffing and resource realities. Participants’ critiques of requirements for multiple nurses per procedure highlight a fundamental mismatch between guideline design and local implementation capacity. This emphasizes that for guidelines to be effective, they must be co-developed or at least adapted in collaboration with frontline staff to reflect staffing realities and resource constraints [37].

Collectively, these findings extend existing literature by demonstrating that in the Tanzanian ICU setting, barriers to ETS guideline adherence are synergistic, with resource scarcity shaping behavioural responses and rendering guideline use aspirational rather than operational. Addressing non-adherence in such contexts, therefore, requires system-level investment, contextual adaptation of guidelines, and institutional support for training rather than strategies focused solely on individual compliance.

### Implications for practice

The findings of this study suggest that a multi-faceted, system-level approach is required to enhance adherence to clinical practice guidelines:

- For hospital management team: Investment in adequate staffing levels, reliable equipment, and functional infrastructure is a foundational requirement for patient safety and adherence to clinical practice guidelines.
- For nurse managers and educators: Training programs focusing on solidifying EBP competencies must be continuous. Furthermore, guidelines must be locally adapted to create realistic, context-specific protocols that nurses in the clinical area can actually follow.

## Strengths and limitations of the study

This study used a semi-structured interview guide, which enhanced consistency across interviews while allowing flexibility to explore emerging issues, thereby strengthening the depth of the findings. However, several methodological limitations need to be acknowledged. Data were collected from a single tertiary referral hospital, which may limit the transferability of the findings to healthcare settings with different resource levels and organizational contexts. Additionally, the use of semi-structured interviews introduces the potential for social desirability bias, as participants may have provided responses perceived as professionally acceptable; this risk was mitigated by conducting interviews in private settings and assuring participants of confidentiality. Furthermore, the study focused exclusively on ICU nurses, excluding other key stakeholders such as physicians, respiratory therapists, and patients whose perspectives could have provided a more comprehensive understanding of the challenges associated with implementing ETS guidelines.

Future research should investigate variation in barriers across demographic and professional lines in the implementation of clinical practice guidelines. A national survey to gather current local statistics on ICU staffing, equipment, and training would provide the foundational data needed for this deeper analysis.

## Conclusion

This study revealed three key barriers to ETS guideline implementation: resource scarcity, human and behavioural challenges, and guideline accessibility and usability issues. Resource barriers, especially inadequate staffing and equipment, were the most critical, directly compromising adherence and patient safety. Non-adherence is therefore less about neglect and more a reflection of systemic constraints, underscoring the need for improved staffing, training, and context-sensitive guideline adaptation.

## Supporting information

S1 Checklist Standards for Reporting Qualitative Research (SRQR) checklist. (DOCX)

S2 Text Interview guide. (DOCX)

S3 Table Excerpts from interviews. (DOCX)

## Abbrevations

EBP: Evidence-Based Practice
ETS: Endotracheal Tube Suctioning
ICUs: Intensive Care Units
MNH: Muhimbili National Hospital
MUHAS: Muhimbili University of Health and Allied Sciences
VAP: Ventilator-Associated Pneumonia.
