# Supplementary material for: Barriers to adherence to endotracheal tube suctioning guidelines among intensive care nurses at a Tanzanian national hospital: A qualitative study
Source: PLoS One. 2026 Jun 16;21(6):e0347186. doi: 10.1371/journal.pone.0347186 (PMC13271520; doi:10.1371/journal.pone.0347186)
Supplement: S2 Text — (DOCX) [file pone.0347186.s002.docx]

Interview guide

**Title: Barriers to adherence to endotracheal tube suctioning guidelines among intensive care nurses at a Tanzanian national hospital: A qualitative study**

Date of interview……………………

Interview site………………………...

Interviewee No ………………………

Start time……………………………

End time………………………………

**A: Interviewee background**

Age of participant………………………………

Sex ……………………………………………..

Work designation……………………………….

Level of education………………………………

Work experience………………………………..

**B: Interview questions**

1. Can you describe your typical experience when performing endotracheal suctioning on a patient in the ICU?
2. What factors or situations have made it difficult for you to apply endotracheal tube suctioning guidelines in your practice?”

***Probes***

- *Can you tell me how staff availability impacted adherence to the guidelines*
- *Can you tell me about any equipment or supply shortages you've faced?*
- *Have you ever encountered situations where the guidelines felt unrealistic?*

1. If you were in a position to change one or two things to make it easier for staff to follow the suctioning guidelines, what would you change?

***Probes***

- *What kind of training or support would be most helpful?*
- *How could the equipment or its availability be improved*?

1. Is there anything else we haven't discussed that you think is important?

**Thank you for your participation**
