# Supplementary material for: Barriers to adherence to endotracheal tube suctioning guidelines among intensive care nurses at a Tanzanian national hospital: A qualitative study
Source: PLoS One. 2026 Jun 16;21(6):e0347186. doi: 10.1371/journal.pone.0347186 (PMC13271520; doi:10.1371/journal.pone.0347186)
Supplement: S3 Table — (DOCX) [file pone.0347186.s003.docx]

**Excerpts from interviews**

| **Subthemes** | **Quotes** |
| --- | --- |
| **Inadequate staffing** | P13: “Science requires two people for suctioning, but in reality, you might be alone. You end up doing something that you think helps, but it may cause problems later.”  P5: “You just shout for help from anyone who is free. You'll even see people coming with an emergency trolley surrounding the patient.”  P10: “With two people, contamination is reduced, and restless patients are easier to manage.”  P6: “It becomes chaotic in that limited space.”  P11: “A blocked tube kills a conscious patient because no doctor assessed it for two weeks.” |
| **Shortage of essential equipment and supplies** | P10: “We improvise by cutting catheters to estimate depth when proper tools are unavailable.”  P11: “We use water for injection when nebulizers are unavailable, despite the risks.”  P3: “We reuse catheters when supplies run out, but clean them with antiseptic, though it's not ideal.”  P5: “The suction kits should be sufficient because if there is a scarcity of those, the patient could acquire infections.”  P2: “In our country, we may not have enough supplies, so you need to finish suctioning with the same catheter.”  P9: “Before closed suction systems, we reused disposable catheters due to shortages. |
