# Supplementary material for: Barriers to adherence to endotracheal tube suctioning guidelines among intensive care nurses at a Tanzanian national hospital: A qualitative study
Source: PLoS One. 2026 Jun 16;21(6):e0347186. doi: 10.1371/journal.pone.0347186 (PMC13271520; doi:10.1371/journal.pone.0347186)
Supplement: Attachment — Submitted filename: Reviewer Report.docx [file pone.0347186.s004.docx]

Dear Editor

I hope this message finds you well. I am writing to provide my feedback on the manuscript ID PONE-D-25-50691, entitled "Barriers to adherence to endotracheal tube suctioning guidelines among intensive care nurses at a Tanzanian national hospital: A qualitative study".

First, I would like to thank the authors for their effort in addressing this important topic.

**General:**

- **English editing is necessary.**

### ****Introduction and Background****

- Link ETS non-adherence to patient outcomes (rates of ventilator-associated pneumonia (VAP) or ICU mortality) to emphasize significance in low-resource settings?
- Compare global adherence rates and challenges to those in resource-limited settings like Tanzania. Highlight differences in staffing, equipment, and guideline dissemination
- The background notes that non-adherence exists and that knowledge deficits have been observed in Tanzania. However the barriers themselves are not sufficiently detailed and the rationale for a qualitative exploratory approach is implied but not explicitly stated.
- Clearly articulate the research gap, including systemic, behavioral, and contextual barriers.
- Justify the qualitative design by emphasizing the need for in-depth, context-specific understanding.
- Include local statistics on ICU staffing ratios, equipment availability, or training frequency.
- Strengthen the study aim by explicitly linking to practical, policy, or clinical implications.

### ****Methods****

- **Participant Recruitment and Sample Size:**
- Briefly describe how saturation was determined (e.g., no new codes or themes emerging after a specific number of interviews).
- State whether there were any refusals or dropouts.
- **Data Collection:**
- Explain how field notes were used in the analysis (e.g., to contextualize verbal responses, capture nonverbal cues).
- **Data analysis:**
- Clarify how disagreements in coding or theme development were resolved.
- **Provide a brief rationale for the final three themes and six subthemes.**

### ****Results****

- Ensure that quotes are evenly distributed across participant responses to avoid over-representing particular individuals. For instance, ensure that perspectives from both Nursing Officers (NOs) and Assistant Nursing Officers (ANOs) are equally reflected.
- Provide a table showing the number of participants who contributed to each theme or subtheme.
- Expanding on how themes were validated. For example, briefly mention if the themes were reviewed by participants or if the analysis was triangulated with other sources to enhance credibility.
- Explicitly link each theme back to the broader research question and its implications for practice. For example, under Resource Barriers, emphasize how the lack of equipment or staff directly compromises adherence to evidence-based ETS practices and patient outcomes.
- Exploring demographic influences and practical implications in greater depth. For example, do less experienced nurses report different challenges compared to more experienced ones? Do Nursing Officers face different barriers than Assistant Nursing Officers? This analysis would add depth to the results.
- Consider explicitly discussing the interplay between themes. For example, how do resource barriers exacerbate human and behavioral challenges, such as resistance to change?

### ****Discussion****

- Apply a more critical comparative approach by emphasizing how the Tanzanian context diverges from previous research findings. In addition, several citations could be more effectively contextualized to illustrate how they specifically inform or contrast with the current study’s results, rather than simply reinforcing them.
- Briefly mention limitations of the methods (e.g., single-site study, potential for social desirability bias in interviews
