# Supplementary material for: Barriers to adherence to endotracheal tube suctioning guidelines among intensive care nurses at a Tanzanian national hospital: A qualitative study
Source: PLoS One. 2026 Jun 16;21(6):e0347186. doi: 10.1371/journal.pone.0347186 (PMC13271520; doi:10.1371/journal.pone.0347186)
Supplement: Attachment — Submitted filename: Response to Reviwer Comments.docx [file pone.0347186.s006.docx]

29/12/ 2025

To

Editor-in-Chief

PLOS One

Dear Sir/Madam,

On behalf of my co-authors, I express our sincere appreciation for the valuable and constructive review of our manuscript entitled “Barriers to adherence to endotracheal tube suctioning guidelines among intensive care nurses at a Tanzanian national hospital: A qualitative study” We have carefully revised the manuscript in line with the reviewers’ comments and provided clarifications where necessary. Additionally, all editorial comments have also been accommodated.

The detailed, point-by-point responses to all reviewer comments are presented in the response sheet that follows this letter.

Yours sincerely,

Menti L. Ndile (Corresponding Author)

Muhimbili University of Health and Allied Sciences

E-mail: [menti.ndile@muhas.ac.tz](mailto:menti.ndile@muhas.ac.tz), mentlast@yahoo.co.uk

**REVIEWER COMMENTS**

**General**

***Comment***: English editing is necessary.

***Response:*** Thank you for this comment. The manuscript has been professionally edited for English language clarity and readability by language editor tools.

**Introduction and Background**

***Comment:*** Link ETS non-adherence to patient outcomes (rates of ventilator-associated pneumonia (VAP) or ICU mortality) to emphasize significance in low-resource settings?

***Response:*** Thank you for this comment. We have revised the Introduction and Background to explicitly link non-adherence to endotracheal suctioning guidelines with adverse patient outcomes, including increased rates of ventilator-associated pneumonia and ICU mortality. These revisions are reflected in lines 61–63 of the revised manuscript

***Comment:*** Compare global adherence rates and challenges to those in resource-limited settings like Tanzania. Highlight differences in staffing, equipment, and guideline dissemination

***Response:*** Thank you for this comment. We have revised the Introduction and Background to compare adherence rates and challenges between high-income and resource-limited settings, highlighting differences in staffing levels, availability of equipment, and dissemination of clinical guidelines. These revisions are reflected in lines 45–53 of the revised manuscript

***Comment:*** The background notes that non-adherence exists and that knowledge deficits have been observed in Tanzania. However the barriers themselves are not sufficiently detailed and the rationale for a qualitative exploratory approach is implied but not explicitly stated.

***Response:*** Thank you for this comment. The Background has been revised to explicitly describe the systemic, behavioral, and contextual barriers to adherence in the Tanzanian ICU setting (lines 45–53). In addition, the rationale for adopting a qualitative exploratory design has been clearly articulated to justify the need for in-depth, context-specific understanding of these barriers (lines 66–70).

***Comment:*** Clearly articulate the research gap, including systemic, behavioral, and contextual barriers. Justify the qualitative design by emphasizing the need for in-depth, context-specific understanding.

***Response:*** Thank you for this comment. The research gap and justification for the qualitative exploratory design have been clearly articulated in the revised manuscript (lines 66–71). Given limited local data, the study emphasizes nurses’ perceptions of systemic, behavioral, and contextual barriers to ETS adherence.

***Comment:*** Include local statistics on ICU staffing ratios, equipment availability, or training frequency.

***Response:*** Some local statistics on ICU status from previous studies have been added-(Lines 54-60). The need for an updated survey on ICU staffing ratio, equipment and training is further suggested for future research for more comprehensive and current data. Lines 334-337

***Comment:*** Strengthen the study aim by explicitly linking to practical, policy, or clinical implications.

**Response:** Thank you for this comment. We have strengthened the study aim by articulating its practical, clinical, and policy implications -Lines 67-71

**Methods**

***Comment:*** Briefly describe how saturation was determined (e.g., no new codes or themes emerging after a specific number of interviews).

***Response:*** Thank you for the comment. The information about the saturation and study participation has been updated and is reflected in lines 89-91

***Comment:*** State whether there were any refusals or dropouts.

***Response:*** Thank you for the comment. There were no refusals or dropouts; this is covered in lines 91-92

***Comment:*** Explain how field notes were used in the analysis (e.g., to contextualize verbal responses, capture nonverbal cues).

***Response:*** Thank you for the comment. The information about how field notes were used in the analysis has been added and is reflected in lines 107-112

**Comment:** Clarify how disagreements in coding or theme development were resolved.

**Response:** Thank you for the comment. The statement about how disagreements were resolved during coding and theme development has been added-Lines 124-126

***Comment:*** Provide a brief rationale for the final three themes and six subthemes.

***Response:*** Thank you for the comment. A brief rationale for the final themes and subthemes has been added-Lines 127-130

**Results**

***Comment:*** Ensure that quotes are evenly distributed across participant responses to avoid over-representing particular individuals. For instance, ensure that perspectives from both Nursing Officers (NOs) and Assistant Nursing Officers (ANOs) are equally reflected

***Response:*** This has been taken into consideration during the reporting of results the result section.

***Comment:*** Provide a table showing the number of participants who contributed to each theme or subtheme.

***Response:*** Thank you for the comment. As this was a qualitative study, the emphasis was placed on depth and richness of data rather than quantification of responses. However, a table indicating the number of participants contributing to each theme and subtheme can be provided as a supplementary file upon journal request.

***Comment:*** Expanding on how themes were validated. For example, briefly mention if the themes were reviewed by participants or if the analysis was triangulated with other sources to enhance credibility.

***Response***: Thank you for the comment. In addition to research team theme validation in the data analysis section, participants were also involved in validation through member checking, as explained in the Trustworthiness section-Line 135-137

***Comment:*** Explicitly link each theme back to the broader research question and its implications for practice. For example, under Resource Barriers, emphasize how the lack of equipment or staff directly compromises adherence to evidence-based ETS practices and patient outcomes.

***Response:*** Thank you for the comment. Considering that this is the Result section, the findings are presented as they are. Linkage of themes to the research question and its implications for practice is captured in the discussion section as an analytical part. Lines 256-312

***Comment:*** Exploring demographic influences and practical implications in greater depth. For example, do less experienced nurses report different challenges compared to more experienced ones? Do Nursing Officers face different barriers than Assistant Nursing Officers? This analysis would add depth to the results.

***Response:*** Thank you for this valuable comment. While the study included participants with varying levels of experience and professional roles, it was not designed to systematically compare demographic subgroups, such as less versus more experienced nurses or Nursing Officers versus Assistant Nursing Officers. As a result, differential barriers across these groups could not be explored. This has been acknowledged as a limitation of the study, and we recommend that future research use mixed-methods approaches to capture the information. Lines 334-337

***Comment:*** Consider explicitly discussing the interplay between themes. For example, how do resource barriers exacerbate human and behavioral challenges, such as resistance to change?

***Response:*** Thank you for the comment. This has been accommodated in the revised Discussion section. Lines 256-312

**Discussion**

**Comment:** Apply a more critical comparative approach by emphasizing how the Tanzanian context diverges from previous research findings. In addition, several citations could be more effectively contextualized to illustrate how they specifically inform or contrast with the current study’s results, rather than simply reinforcing them.

**Response:** Thank you for this insightful comment. The Discussion section has been revised to adopt a more critical comparative approach, explicitly highlighting how the Tanzanian ICU context diverges from findings reported in previous studies, particularly those conducted in higher-resource settings. Lines 256-312

***Comment:*** Briefly mention limitations of the methods (e.g., single-site study, potential for social desirability bias in interviews

***Response:*** Thank you for the comment. This has been acknowledged in the revised Strengths and Limitations section. Lines 325-330
