# Supplementary material for: Barriers to adherence to endotracheal tube suctioning guidelines among intensive care nurses at a Tanzanian national hospital: A qualitative study
Source: PLoS One. 2026 Jun 16;21(6):e0347186. doi: 10.1371/journal.pone.0347186 (PMC13271520; doi:10.1371/journal.pone.0347186)
Supplement: Peer review history

# Peer review: Decision Letter 0

**DOI:** 10.1371/journal.pone.0347186.r001

**Authors:** Katrien Janin

25 Nov 2025

PONE-D-25-50691

Barriers to adherence to endotracheal tube suctioning guidelines among intensive care nurses at a Tanzanian national hospital: A qualitative study

PLOS ONE

Dear Dr. Ndile,

Thank you for submitting your manuscript to PLOS ONE. After careful consideration, we feel that it has merit but does not fully meet PLOS ONE’s publication criteria as it currently stands. Therefore, we invite you to submit a revised version of the manuscript that addresses the points raised during the review process.

Please submit your revised manuscript by Jan 09 2026 11:59PM. If you will need more time than this to complete your revisions, please reply to this message or contact the journal office at plosone@plos.org. When you’re ready to submit your revision, log on to https://www.editorialmanager.com/pone/ and select the ‘Submissions Needing Revision’ folder to locate your manuscript file.

Please include the following items when submitting your revised manuscript:

- A rebuttal letter that responds to each point raised by the academic editor and reviewer(s). You should upload this letter as a separate file labeled ‘Response to Reviewers’.
- A marked-up copy of your manuscript that highlights changes made to the original version. You should upload this as a separate file labeled ‘Revised Manuscript with Track Changes’.
- An unmarked version of your revised paper without tracked changes. You should upload this as a separate file labeled ‘Manuscript’.

If you would like to make changes to your financial disclosure, please include your updated statement in your cover letter. Guidelines for resubmitting your figure files are available below the reviewer comments at the end of this letter.

If applicable, we recommend that you deposit your laboratory protocols in protocols.io to enhance the reproducibility of your results. Protocols.io assigns your protocol its own identifier (DOI) so that it can be cited independently in the future. For instructions see: https://journals.plos.org/plosone/s/submission-guidelines#loc-laboratory-protocols. Additionally, PLOS ONE offers an option for publishing peer-reviewed Lab Protocol articles, which describe protocols hosted on protocols.io. Read more information on sharing protocols at https://plos.org/protocols?utm_medium=editorial-email&utm_source=authorletters&utm_campaign=protocols.

We look forward to receiving your revised manuscript.

Kind regards,

Katrien G. Janin, PhD

Staff Editor

PLOS ONE

Journal Requirements:

When submitting your revision, we need you to address these additional requirements.

1.Please ensure that your manuscript meets PLOS ONE’s style requirements, including those for file naming. The PLOS ONE style templates can be found at

https://journals.plos.org/plosone/s/file?id=wjVg/PLOSOne_formatting_sample_main_body.pdf and

https://journals.plos.org/plosone/s/file?id=ba62/PLOSOne_formatting_sample_title_authors_affiliations.pdf

2. We are unable to open your Supporting Information file “Interview guide.docx and Excerpts from transcripts.docx”. Please kindly revise as necessary and re-upload.

3. If the reviewer comments include a recommendation to cite specific previously published works, please review and evaluate these publications to determine whether they are relevant and should be cited. There is no requirement to cite these works unless the editor has indicated otherwise.

Additional Editor Comments:

-->The manuscript has been evaluated by two reviewers, and their comments are available below.

The reviewers have raised a number of concerns. They feel request you include local statistics on ICU staffing ratios, equipment availability, and/or training frequency, and like to you to strengthen the study aim by explicitly linking to practical, policy, or clinical implications.

Upon internal evaluation of the review s provided, we kindly request you to disregard the reviewer report provided by Reviewer 2. No amendments are required in response to reviewer 2’s comments"

Could you please carefully revise the manuscript to address all comments raised by Reviewer 1?

[Note: HTML markup is below. Please do not edit.]

Reviewer’s Responses to Questions

**Comments to the Author**

1. Is the manuscript technically sound, and do the data support the conclusions?

The manuscript must describe a technically sound piece of scientific research with data that supports the conclusions. Experiments must have been conducted rigorously, with appropriate controls, replication, and sample sizes. The conclusions must be drawn appropriately based on the data presented.

Reviewer #1: Yes

Reviewer #2: Yes

**********

2. Has the statistical analysis been performed appropriately and rigorously?

Reviewer #1: N/A

Reviewer #2: Yes

**********

3. Have the authors made all data underlying the findings in their manuscript fully available?

The PLOS Data policy requires authors to make all data underlying the findings described in their manuscript fully available without restriction, with rare exception (please refer to the Data Availability Statement in the manuscript PDF file). The data should be provided as part of the manuscript or its supporting information, or deposited to a public repository. For example, in addition to summary statistics, the data points behind means, medians and variance measures should be available. If there are restrictions on publicly sharing data—e.g. participant privacy or use of data from a third party—those must be specified.

Reviewer #1: No

Reviewer #2: Yes

**********

4. Is the manuscript presented in an intelligible fashion and written in standard English?

PLOS ONE does not copyedit accepted manuscripts, so the language in submitted articles must be clear, correct, and unambiguous. Any typographical or grammatical errors should be corrected at revision, so please note any specific errors here.

Reviewer #1: Yes

Reviewer #2: Yes

**********

5. Review Comments to the Author

Please use the space provided to explain your answers to the questions above. You may also include additional comments for the author, including concerns about dual publication, research ethics, or publication ethics. (Please upload your review as an attachment if it exceeds 20,000 characters)

Reviewer #1: First, I would like to thank the authors for their effort in addressing this important topic.

General:

• English editing is necessary.

Introduction and Background:

• Link ETS non-adherence to patient outcomes (rates of ventilator-associated pneumonia (VAP) or ICU mortality) to emphasize significance in low-resource settings?

• Compare global adherence rates and challenges to those in resource-limited settings like Tanzania. Highlight differences in staffing, equipment, and guideline dissemination

• The background notes that non-adherence exists and that knowledge deficits have been observed in Tanzania. However the barriers themselves are not sufficiently detailed and the rationale for a qualitative exploratory approach is implied but not explicitly stated.

o Clearly articulate the research gap, including systemic, behavioral, and contextual barriers.

o Justify the qualitative design by emphasizing the need for in-depth, context-specific understanding.

• Include local statistics on ICU staffing ratios, equipment availability, or training frequency.

• Strengthen the study aim by explicitly linking to practical, policy, or clinical implications.

Methods:

• Participant Recruitment and Sample Size:

o Briefly describe how saturation was determined (e.g., no new codes or themes emerging after a specific number of interviews).

o State whether there were any refusals or dropouts.

• Data Collection:

o Explain how field notes were used in the analysis (e.g., to contextualize verbal responses, capture nonverbal cues).

• Data analysis:

o Clarify how disagreements in coding or theme development were resolved.

o Provide a brief rationale for the final three themes and six subthemes.

Results:

• Ensure that quotes are evenly distributed across participant responses to avoid over-representing particular individuals. For instance, ensure that perspectives from both Nursing Officers (NOs) and Assistant Nursing Officers (ANOs) are equally reflected.

• Provide a table showing the number of participants who contributed to each theme or subtheme.

• Expanding on how themes were validated. For example, briefly mention if the themes were reviewed by participants or if the analysis was triangulated with other sources to enhance credibility.

• Explicitly link each theme back to the broader research question and its implications for practice. For example, under Resource Barriers, emphasize how the lack of equipment or staff directly compromises adherence to evidence-based ETS practices and patient outcomes.

• Exploring demographic influences and practical implications in greater depth. For example, do less experienced nurses report different challenges compared to more experienced ones? Do Nursing Officers face different barriers than Assistant Nursing Officers? This analysis would add depth to the results.

• Consider explicitly discussing the interplay between themes. For example, how do resource barriers exacerbate human and behavioral challenges, such as resistance to change?

Discussion:

• Apply a more critical comparative approach by emphasizing how the Tanzanian context diverges from previous research findings. In addition, several citations could be more effectively contextualized to illustrate how they specifically inform or contrast with the current study’s results, rather than simply reinforcing them.

• Briefly mention limitations of the methods (e.g., single-site study, potential for social desirability bias in interviews

Reviewer #2: Buyi et al. present a well-structured qualitative study exploring barriers to adherence to endotracheal tube suctioning (ETS) guidelines among ICU nurses at a Tanzanian national hospital. The authors employ semi-structured interviews and thematic analysis to identify three key themes: resource barriers, human and behavioral challenges, and guideline accessibility and usability. The study is timely and relevant, especially for improving evidence-based nursing practices in resource-limited settings.

The manuscript is well written and methodologically sound, but I have several comments regarding its enhancement of the study’s transparency, credibility, and replicability.

Background

The authors provide a clear and compelling rationale for the study. The problem statement is well articulated, the authors describe endotracheal tube suctioning (ETS) as a critical procedure in ICU settings, emphasizing its role in maintaining airway patency and preventing complications like ventilator-associated pneumonia (VAP). The research gap clearly states that while non-adherence to ETS guidelines is recognized, the specific barriers in the Tanzanian ICU context remain underexplored. The author also reviews existing literature on ETS guidelines and adherence challenges, citing global studies that document suboptimal compliance.

Purpose or research question

The purpose of the study is clearly stated: to explore the barriers to ETS guideline adherence among ICU nurses at Muhimbili National Hospital (MNH), Tanzania. However, the article did not mention specific research questions that guide the inquiry and clarify its objectives.

Methods

The use of an exploratory qualitative design is appropriate. The authors followed Braun and Clarke’s thematic analysis framework, which is suitable and well executed. The Standards for Reporting Qualitative Research (SRQR) in the manuscript is generally well-structured and addresses many key elements. However, several components are either missing or underreported, which could affect the transparency and completeness of the study.

Moreover, the manuscript lacks sufficient detail on researcher characteristics and reflexivity. The reflexive journaling is briefly mentioned, there is no clear description of the interviewer’s background, gender, training, or relationship to participants. This information is essential to understanding potential biases and the influence of the researcher on data collection and interpretation. Given the hierarchical nature of hospital settings, potential power dynamics should be acknowledged and discussed. This would enhance transparency and help readers assess the influence of the researcher on data collection and interpretation.

Participant recruitment and sample size

Purposive sampling was used, and the sample size (17) was justified through data saturation. The demographic table is clear and informative. However, the manuscript does not mention whether any participants declined to participate or dropped out. This should be clarified.

Additionally, while sampling saturation is mentioned, the process used to determine saturation whether it was code saturation or meaning saturation is not explained. Clarifying this would strengthen the justification for the sample size.

Data Collection

Semi-structured interviews were conducted in Kiswahili, audio-recorded, and transcribed verbatim. Field notes were taken, and member checking was performed. These are strengths. However, the manuscript does not specify whether repeat interviews were conducted, nor does it mention the presence or absence of non-participants during interviews. These details should be added.

Data Analysis

The inductive thematic analysis is rigorous and well described. Collaborative coding and bilingual verification of translations strengthen the validity of findings. However, the manuscript does not mention whether any qualitative data analysis software (e.g., NVivo, Atlas.ti) was used. Including this information would enhance methodological transparency.

Findings

Themes and subthemes are clearly presented and supported by rich participant quotations. The discussion is well integrated with existing literature and reflects the realities of the study context. The conclusions are consistent with the data and offer practical recommendations.

Discussion

The manuscript addresses several key elements expected in qualitative research reporting. The authors begin with a clear summary of the main findings, identifying three thematic barriers to guideline adherence, and effectively connect the results to existing literature, showing how their findings support and expand upon previous studies. However, while they acknowledge the study’s limited generalizability, the discussion of broader applicability could be more developed. The manuscript does identify its unique contribution by highlighting the systemic nature of non-adherence in a Tanzanian ICU context, which adds valuable insight to the field of nursing practice in resource-limited settings. Overall, the discussion is coherent and well-integrated, though it could benefit from a more explicit articulation of theoretical implications and potential for policy or practice impact beyond the study site.

Ethical Considerations

Ethical approval and informed consent procedures are clearly described. Participants were informed of their rights, and confidentiality was maintained. This section is well handled.

Data Availability

The authors confirm that all relevant data are available within the manuscript and supporting files, including the interview guide and transcript excerpts. This satisfies journal requirements.

Presentation and Language

The manuscript is written in standard English and is easy to follow. Minor typographical and formatting issues should be corrected during revision.

I recommend minor revisions for this manuscript to improve reporting clarity, researcher reflexivity, and alignment with the SRQR checklist.

**********

6. PLOS authors have the option to publish the peer review history of their article (what does this mean?). If published, this will include your full peer review and any attached files.

If you choose “no”, your identity will remain anonymous but your review may still be made public.

**Do you want your identity to be public for this peer review?**  For information about this choice, including consent withdrawal, please see our Privacy Policy.

Reviewer #1: No

Reviewer #2: No

**********

[NOTE: If reviewer comments were submitted as an attachment file, they will be attached to this email and accessible via the submission site. Please log into your account, locate the manuscript record, and check for the action link “View Attachments”. If this link does not appear, there are no attachment files.]

To ensure your figures meet our technical requirements, please review our figure guidelines: https://journals.plos.org/plosone/s/figures

You may also use PLOS’s free figure tool, NAAS, to help you prepare publication quality figures: https://journals.plos.org/plosone/s/figures#loc-tools-for-figure-preparation.

NAAS will assess whether your figures meet our technical requirements by comparing each figure against our figure specifications.

Attachment Submitted filename: Reviewer Report.docx

# Author comment: Author response to Decision Letter 1

**DOI:** 10.1371/journal.pone.0347186.r002

30 Dec 2025

REVIEWER COMMENTS AND RESPONSE

General

Comment: English editing is necessary.

Response: Thank you for this comment. The manuscript has been professionally edited for English language clarity and readability by language editor tools.

Introduction and Background

Comment: Link ETS non-adherence to patient outcomes (rates of ventilator-associated pneumonia (VAP) or ICU mortality) to emphasize significance in low-resource settings?

Response: Thank you for this comment. We have revised the Introduction and Background to explicitly link non-adherence to endotracheal suctioning guidelines with adverse patient outcomes, including increased rates of ventilator-associated pneumonia and ICU mortality. These revisions are reflected in lines 61–63 of the revised manuscript

Comment: Compare global adherence rates and challenges to those in resource-limited settings like Tanzania. Highlight differences in staffing, equipment, and guideline dissemination

Response: Thank you for this comment. We have revised the Introduction and Background to compare adherence rates and challenges between high-income and resource-limited settings, highlighting differences in staffing levels, availability of equipment, and dissemination of clinical guidelines. These revisions are reflected in lines 45–53 of the revised manuscript

Comment: The background notes that non-adherence exists and that knowledge deficits have been observed in Tanzania. However the barriers themselves are not sufficiently detailed and the rationale for a qualitative exploratory approach is implied but not explicitly stated.

Response: Thank you for this comment. The Background has been revised to explicitly describe the systemic, behavioral, and contextual barriers to adherence in the Tanzanian ICU setting (lines 45–53). In addition, the rationale for adopting a qualitative exploratory design has been clearly articulated to justify the need for in-depth, context-specific understanding of these barriers (lines 66–70).

Comment: Clearly articulate the research gap, including systemic, behavioral, and contextual barriers. Justify the qualitative design by emphasizing the need for in-depth, context-specific understanding.

Response: Thank you for this comment. The research gap and justification for the qualitative exploratory design have been clearly articulated in the revised manuscript (lines 66–71). Given limited local data, the study emphasizes nurses’ perceptions of systemic, behavioral, and contextual barriers to ETS adherence.

Comment: Include local statistics on ICU staffing ratios, equipment availability, or training frequency.

Response: Some local statistics on ICU status from previous studies have been added-(Lines 54-60). The need for an updated survey on ICU staffing ratio, equipment and training is further suggested for future research for more comprehensive and current data. Lines 334-337

Comment: Strengthen the study aim by explicitly linking to practical, policy, or clinical implications.

Response: Thank you for this comment. We have strengthened the study aim by articulating its practical, clinical, and policy implications -Lines 67-71

Methods

Comment: Briefly describe how saturation was determined (e.g., no new codes or themes emerging after a specific number of interviews).

Response: Thank you for the comment. The information about the saturation and study participation has been updated and is reflected in lines 89-91

Comment: State whether there were any refusals or dropouts.

Response: Thank you for the comment. There were no refusals or dropouts; this is covered in lines 91-92

Comment: Explain how field notes were used in the analysis (e.g., to contextualize verbal responses, capture nonverbal cues).

Response: Thank you for the comment. The information about how field notes were used in the analysis has been added and is reflected in lines 107-112

Comment: Clarify how disagreements in coding or theme development were resolved.

Response: Thank you for the comment. The statement about how disagreements were resolved during coding and theme development has been added-Lines 124-126

Comment: Provide a brief rationale for the final three themes and six subthemes.

Response: Thank you for the comment. A brief rationale for the final themes and subthemes has been added-Lines 127-130

Results

Comment: Ensure that quotes are evenly distributed across participant responses to avoid over-representing particular individuals. For instance, ensure that perspectives from both Nursing Officers (NOs) and Assistant Nursing Officers (ANOs) are equally reflected

Response: This has been taken into consideration during the reporting of results the result section.

Comment: Provide a table showing the number of participants who contributed to each theme or subtheme.

Response: Thank you for the comment. As this was a qualitative study, the emphasis was placed on depth and richness of data rather than quantification of responses. However, a table indicating the number of participants contributing to each theme and subtheme can be provided as a supplementary file upon journal request.

Comment: Expanding on how themes were validated. For example, briefly mention if the themes were reviewed by participants or if the analysis was triangulated with other sources to enhance credibility.

Response: Thank you for the comment. In addition to research team theme validation in the data analysis section, participants were also involved in validation through member checking, as explained in the Trustworthiness section-Line 135-137

Comment: Explicitly link each theme back to the broader research question and its implications for practice. For example, under Resource Barriers, emphasize how the lack of equipment or staff directly compromises adherence to evidence-based ETS practices and patient outcomes.

Response: Thank you for the comment. Considering that this is the Result section, the findings are presented as they are. Linkage of themes to the research question and its implications for practice is captured in the discussion section as an analytical part. Lines 256-312

Comment: Exploring demographic influences and practical implications in greater depth. For example, do less experienced nurses report different challenges compared to more experienced ones? Do Nursing Officers face different barriers than Assistant Nursing Officers? This analysis would add depth to the results.

Response: Thank you for this valuable comment. While the study included participants with varying levels of experience and professional roles, it was not designed to systematically compare demographic subgroups, such as less versus more experienced nurses or Nursing Officers versus Assistant Nursing Officers. As a result, differential barriers across these groups could not be explored. This has been acknowledged as a limitation of the study, and we recommend that future research use mixed-methods approaches to capture the information. Lines 334-337

Comment: Consider explicitly discussing the interplay between themes. For example, how do resource barriers exacerbate human and behavioral challenges, such as resistance to change?

Response: Thank you for the comment. This has been accommodated in the revised Discussion section. Lines 256-312

Discussion

Comment: Apply a more critical comparative approach by emphasizing how the Tanzanian context diverges from previous research findings. In addition, several citations could be more effectively contextualized to illustrate how they specifically inform or contrast with the current study’s results, rather than simply reinforcing them.

Response: Thank you for this insightful comment. The Discussion section has been revised to adopt a more critical comparative approach, explicitly highlighting how the Tanzanian ICU context diverges from findings reported in previous studies, particularly those conducted in higher-resource settings. Lines 256-312

Comment: Briefly mention limitations of the methods (e.g., single-site study, potential for social desirability bias in interviews

Response: Thank you for the comment. This has been acknowledged in the revised Strengths and Limitations section. Lines 325-330

Attachment Submitted filename: Response to Reviwer Comments.docx

# Peer review: Decision Letter 1

**DOI:** 10.1371/journal.pone.0347186.r003

**Authors:** Marianne Clemence

6 May 2026

Barriers to adherence to endotracheal tube suctioning guidelines among intensive care nurses at a Tanzanian national hospital: A qualitative study

PONE-D-25-50691R1

Dear Dr. Ndile,

We’re pleased to inform you that your manuscript has been judged scientifically suitable for publication and will be formally accepted for publication once it meets all outstanding technical requirements.

Within one week, you’ll receive an e-mail detailing the required amendments. When these have been addressed, you’ll receive a formal acceptance letter and your manuscript will be scheduled for publication.

An invoice will be generated when your article is formally accepted. Please note, if your institution has a publishing partnership with PLOS and your article meets the relevant criteria, all or part of your publication costs will be covered. Please make sure your user information is up-to-date by logging into Editorial Manager at Editorial Manager® and clicking the ‘Update My Information’ link at the top of the page. For questions related to billing, please contact billing support.

If your institution or institutions have a press office, please notify them about your upcoming paper to help maximize its impact. If they’ll be preparing press materials, please inform our press team as soon as possible -- no later than 48 hours after receiving the formal acceptance. Your manuscript will remain under strict press embargo until 2 pm Eastern Time on the date of publication. For more information, please contact onepress@plos.org.

Kind regards,

Marianne Clemence

Staff Editor

PLOS One

Additional Editor Comments (optional):

Please note that it is not necessary to address the comments of Reviewer 3 to meet our publication criteria.

Reviewers’ comments:

Reviewer’s Responses to Questions

**Comments to the Author**

1. If the authors have adequately addressed your comments raised in a previous round of review and you feel that this manuscript is now acceptable for publication, you may indicate that here to bypass the “Comments to the Author” section, enter your conflict of interest statement in the “Confidential to Editor” section, and submit your “Accept” recommendation.

Reviewer #2: All comments have been addressed

Reviewer #3: (No Response)

Reviewer #4: All comments have been addressed

**********

2. Is the manuscript technically sound, and do the data support the conclusions?

The manuscript must describe a technically sound piece of scientific research with data that supports the conclusions. Experiments must have been conducted rigorously, with appropriate controls, replication, and sample sizes. The conclusions must be drawn appropriately based on the data presented.

Reviewer #2: Yes

Reviewer #3: Yes

Reviewer #4: Yes

**********

3. Has the statistical analysis been performed appropriately and rigorously?

Reviewer #2: Yes

Reviewer #3: Yes

Reviewer #4: Yes

**********

4. Have the authors made all data underlying the findings in their manuscript fully available?

The PLOS Data policy requires authors to make all data underlying the findings described in their manuscript fully available without restriction, with rare exception (please refer to the Data Availability Statement in the manuscript PDF file). The data should be provided as part of the manuscript or its supporting information, or deposited to a public repository. For example, in addition to summary statistics, the data points behind means, medians and variance measures should be available. If there are restrictions on publicly sharing data—e.g. participant privacy or use of data from a third party—those must be specified.

Reviewer #2: Yes

Reviewer #3: Yes

Reviewer #4: Yes

**********

5. Is the manuscript presented in an intelligible fashion and written in standard English?

PLOS ONE does not copyedit accepted manuscripts, so the language in submitted articles must be clear, correct, and unambiguous. Any typographical or grammatical errors should be corrected at revision, so please note any specific errors here.

Reviewer #2: Yes

Reviewer #3: Yes

Reviewer #4: Yes

**********

6. Review Comments to the Author

Please use the space provided to explain your answers to the questions above. You may also include additional comments for the author, including concerns about dual publication, research ethics, or publication ethics. (Please upload your review as an attachment if it exceeds 20,000 characters)

Reviewer #2: (No Response)

Reviewer #3: Manuscript Title

Barriers to adherence to endotracheal tube suctioning guidelines among intensive care nurses at a Tanzanian national hospital: A qualitative study

Major Comments (Minor Revisions Needed)

1. Redundancy and Editing Issues

There are visible duplications and formatting artifacts (likely from track changes):

Example:

“Endotracheal tube suctioning (ETS) is a frequently performed… Endotracheal tube suctioning (ETS) is among the most frequently performed…”

Recommendation:

Remove duplicated sentences in Background section.

Carefully clean manuscript for:

Repeated phrases

Track-change remnants

Formatting inconsistencies

2. Clarity of Abstract (Minor Language Issue)

3. Consistency in Terminology

Some inconsistencies observed:

“Human & behavioural challenges” vs “Human and behavioral challenges”

“Guideline accessibility & usability” vs “Guideline accessibility and usability”

Recommendation:

Use consistent terminology throughout (prefer journal style: and instead of &)

4. Quantification Concern in Qualitative Study

In response to reviewer:

Table of number of participants per theme not included

While justification is valid, journals like PLOS ONE often accept light quantification.

Recommendation (optional but strong):

Add supplementary table showing:

Number of participants contributing to each theme/subtheme

This will strengthen transparency and credibility

5. Link Between Results and Implications

Although addressed in discussion, some sections of results remain purely descriptive.

Suggest minor enhancement:

Add one linking sentence per theme indicating:

“This has implications for patient safety/practice…”

6. Language Polishing (Minor)

Some sentences are still slightly verbose or repetitive.

Example:

“This study revealed three key barriers… Resource barriers… were the most critical…”

Minor Comments

1. Methods Section

Good description of saturation, but could be sharper:

Add: “No new codes/themes emerged after the 15th interview…” (if applicable)

2. Ethics Section

Well written, but could be condensed slightly for brevity.

3. References

Minor formatting inconsistencies:

Spacing issues

Duplicate citation numbering style in some places

Ensure strict PLOS ONE referencing format

4. Tables

Table 2 is good but could be improved:

Align bullets consistently

Ensure formatting uniformity

Overall Recommendation

Minor Revision (close to acceptance)

The manuscript is well-structured, methodologically sound, and addresses an important gap in ICU nursing practice in resource-limited settings. Reviewer comments have been adequately addressed in most sections. Only minor refinements in clarity, consistency, and presentation are required.

Reviewer #4: Thank you for addressing all the comments so thoroughly. The paper has been greatly improved by the additions and amendments made in response to the review. Well done. An important paper that adds to the body of knowledge.

**********

7. PLOS authors have the option to publish the peer review history of their article (what does this mean?). If published, this will include your full peer review and any attached files.

If you choose “no”, your identity will remain anonymous but your review may still be made public.

**Do you want your identity to be public for this peer review?**  For information about this choice, including consent withdrawal, please see our Privacy Policy.

Reviewer #2: **Yes:** Junaidy Suparman Rustam

Reviewer #3: **Yes:** Noorul Amin

Reviewer #4: **Yes:** Angela Flynn

**********

# Editor report: Acceptance letter

**DOI:** 10.1371/journal.pone.0347186.r004

**Authors:** Marianne Clemence

PONE-D-25-50691R1

PLOS One

Dear Dr. Ndile,

I’m pleased to inform you that your manuscript has been deemed suitable for publication in PLOS One. Congratulations! Your manuscript is now being handed over to our production team.

At this stage, our production department will prepare your paper for publication. This includes ensuring the following:

* All references, tables, and figures are properly cited

* All relevant supporting information is included in the manuscript submission,

* There are no issues that prevent the paper from being properly typeset

You will receive further instructions from the production team, including instructions on how to review your proof when it is ready. Please keep in mind that we are working through a large volume of accepted articles, so please give us a few days to review your paper and let you know the next and final steps.

Lastly, if your institution or institutions have a press office, please let them know about your upcoming paper now to help maximize its impact. If they’ll be preparing press materials, please inform our press team within the next 48 hours. Your manuscript will remain under strict press embargo until 2 pm Eastern Time on the date of publication. For more information, please contact onepress@plos.org.

You will receive an invoice from PLOS for your publication fee after your manuscript has reached the completed accept phase. If you receive an email requesting payment before acceptance or for any other service, this may be a phishing scheme. Learn how to identify phishing emails and protect your accounts at https://explore.plos.org/phishing.

If we can help with anything else, please email us at customercare@plos.org.

Thank you for submitting your work to PLOS ONE and supporting open access.

Kind regards,

PLOS ONE Editorial Office Staff

on behalf of

Dr Marianne Clemence

Staff Editor

PLOS One
